# Repetition velocity during the leg and chest press in older adults: influence of starting execution technique

**DOI:** 10.1186/s13102-025-01326-9

**Published:** 2025-09-25

**Authors:** Tiago Sousa, Ana Pereira, Diogo Luís Marques, Henrique Pereira Neiva, David Rodríguez-Rosell, Daniel Almeida Marinho, Mário Cardoso Marques

**Affiliations:** 1 Department of Sport Sciences, University of Beira Interior, Covilhã, 6201-001 Portugal; 2 Instituto Politécnico de Setúbal, Escola Superior de Educação, Setúbal, Portugal; 3 Sport, Physical Activity and Health Research and Innovation Center (SPRINT), Rio Maior, Portugal; 4 Life Quality Research Centre, Rio Maior, 2040-413 Portugal; 5 Research Center in Sports Sciences, Health Sciences, and Human Development (CIDESD), Covilhã, 6201-001 Portugal; 6 Physical Performance and Sports Research Center, Pablo de Olavide University, Seville, Spain; 7 Department of Sport and Informatics, Universidad Pablo de Olavide, Seville, Spain

**Keywords:** Strength training, Execution technique, Movement velocity, Linear transducer, Repetitions, Older people

## Abstract

**Background:**

Effectively implementing velocity-monitored resistance training (RT) requires selecting appropriate methodologies, particularly in older adults. The current study compares the differences in repetition velocity patterns with two different starting execution techniques - eccentric-concentric vs. concentric-only - during RT sets in the leg press (LP) and chest press (CP) in older adults.

**Methods:**

Eighteen participants (67% female; 79 ± 10 years) underwent a 6-week intervention, with the first two weeks dedicated to familiarization and load-velocity profiling. From weeks 3 to 6, participants completed two weekly RT sessions, performing two sets of ten repetitions at 40, 50, 60, and 70% of their one-repetition maximum (1RM), only differing in the starting execution techniques.

**Results:**

The eccentric-concentric technique resulted in higher mean velocity values during the first repetition compared to the concentric-only technique across all relative loads in the LP (average difference: 0.09 ± 0.07 m·s^− 1^; *p* < 0.001; *g* = 0.84) and CP (average difference: 0.07 ± 0.06 m·s^− 1^; *p* < 0.001; *g* = 0.70). No differences were found between execution techniques in the fastest repetition of the set (all *p* > 0.05) and the mean velocity of almost all subsequent repetitions throughout the set. The pattern of repetition velocity was more stable with the eccentric-concentric technique (R^2^: 0.78–0.97) than with the concentric-only technique (R^2^: 0.01–0.52). The fastest repetition typically occurred in the second repetition (67.5%) under the concentric-only technique, but in the first repetition (42%) under the eccentric-concentric technique.

**Conclusions:**

These findings indicate that the initial execution technique adopted in resistance machines significantly impacts the mean velocity of the first repetition among older adults, with higher velocities reached using the eccentric-concentric technique. Therefore, velocity-based RT protocols for older adults should account for starting execution techniques in machine exercises to optimize load monitoring and training prescription effectiveness.

## Introduction

The aging process leads to a significant decrease in the ability to produce force rapidly, primarily due to the atrophy of fast-twitch fibers [1] and a loss of motor units [2], resulting in a decline in functional capacity and an increased risk of falls [3, 4]. Hence, a strong need exists to create strategies that promote healthy aging [5, 6]. Resistance training (RT) is highly recognized as an effective method to mitigate age-related strength losses since it induces neuromuscular adaptations, promoting older adults’ health-related quality of life [3, 7, 8]. The manipulation of the acute RT variables plays a crucial role in these adaptations [9], with volume, relative load, and movement velocity being among the most relevant [10–12].

Recent evidence suggests that coaches can efficiently implement training prescriptions for older adults using a velocity-monitored RT approach [13–15]. Contrary to the typically established procedures, coaches do not prescribe training load and volume based on a predetermined relative load and number of repetitions [16, 17]. Instead, the relative load prescription should be determined based on a specific initial velocity within the set, given the nearly perfect relationship that exists between velocity and relative load (% of one repetition maximum [1RM]) [10, 15]. The individualized load-velocity relationships enable coaches and researchers to prescribe relative load or intensity during resistance training by monitoring movement velocity and assessing the day-to-day effects of each training session in real time [13, 18]. It is essential to recognize that this relationship is affected by a multitude of factors, including, but not limited to, the type of exercise, the individual’s age, and the equipment employed [18, 19]. Conversely, for training volume, the prescription targets a specific magnitude of velocity loss (i.e., the difference between the fastest repetition, usually the first, and the slowest/last repetition) during the set, which, combined with a given relative intensity, serves to determine the number of repetitions performed and consequently the level of effort [10, 11]. Following the velocity-monitored RT approach and its manipulation of the acute training variables that determine the effort performed, researchers typically assume that the first repetition is usually the fastest [11, 16, 20]. This early identification enables understanding if the participant is training according to the programmed relative load. If not, the coach can decide to adjust the weight to match the intended relative load. However, research with strength-trained men demonstrated that, depending on the methods employed, the first repetition may not always be the fastest [12]. Similarly, some evidence on velocity-monitored RT with older adults showed that, on average, the second repetition is usually the fastest in the set, particularly when using resistance machines like the horizontal leg press (LP) and seated chest press (CP) [13–15]. A possible reason for these results in resistance machines could be related to the initial execution technique (concentric-only), which makes it more challenging to overcome the “sticking point” at the start of the movement [21, 22], leading to lower velocity outputs in the first repetition of the set. These observations in resistance machines can pose a challenge for the rigorous implementation of velocity-monitored RT in older adults, as the delay in identifying the programmed relative load can also impact the control of the established intra-set relative velocity loss (e.g., 10% or 20%), thus compromising the validity of using this approach in geriatric settings.

In this sense, planning to start the movement with the eccentric-concentric technique in these resistance machine exercises might be a valid alternative execution condition, as it appears to significantly enhance force production in adults and older adults, differentiating from the conventional concentric-only approach [8, 23]. Thus, performing an eccentric muscular action before the concentric contraction (i.e., a stretch-shortening cycle) can decisively influence the movement’s kinematics [21, 24, 25]. Several underlying factors may contribute to this mechanical improvement, including pre-tension mechanisms, elastic energy storage, and the enhancement of residual force [26–29]. It is important to note that the duration of the transition between eccentric and concentric contractions significantly impacts the effectiveness of the stretch-shortening cycle [27, 30]. For example, for jumps, research shows that an amortization phase of less than 250 ms (ground contact time) indicates a fast stretch-shortening cycle, while movements greater than 250 ms are considered slow; a prolonged amortization phase significantly diminishes the efficacy of the stretch-shortening cycle [27, 30, 31].

This aspect gains particular relevance in the older population, as neuromuscular pre-activation helps counteract the decline in concentric function, causing higher velocity outputs [8]. However, the actual influence of these techniques on the pattern of repetition velocity throughout the RT set among the older population remains unclear. Therefore, given the lack of studies on this topic, it is essential to investigate the potential differences between starting execution techniques – eccentric-concentric vs. concentric-only – in the pattern of repetition velocity over the set, particularly with a focus on the velocity of the first repetition, to enhance the practicality of using a velocity-monitored RT approach with older adults. This analysis will provide relevant practical implications regarding controlling movement velocity in the LP and CP for older people because the sooner the fastest repetition is identified, the better it will be for determining the relative load (i.e., knowing whether the participant is training according to the programmed relative load) and consequently controlling the velocity loss throughout the RT set. Furthermore, given the unique characteristics of the older population, such as lower concentric velocities and a narrower range of movement velocities associated with the relative loads when compared to younger populations, it seems pertinent to investigate the most effective methodologies for implementing this approach within this specific population.

Given the abovementioned considerations, this study analyzed how starting execution techniques – eccentric-concentric vs. concentric-only – affect the pattern of repetition velocity during RT sets in the LP and CP, with a range of loads typically used in RT for older adults. Firstly, we hypothesized that the concentric-only technique would result in lower velocity in the first repetition due to the greater difficulty associated with successfully executing the repetition with this technique. In contrast, the eccentric-concentric technique would produce a higher velocity output in the first repetition due to the stretch-shortening cycle. Finally, we would expect no differences between the execution techniques in terms of the fastest mean velocity (MV) within the set.

## Methods

### Participants

We recruited eighteen older adults (6 men and 12 women; age: 79 ± 10 years; body mass: 64 ± 16 kg; height: 150 ± 11 cm) from residential care facilities. Inclusion criteria comprised individuals aged ≥ 60, able to collaborate with the team and to perform the selected exercises, with a resistance training background, having performed the LP and CP for at least six months. The study excluded individuals with severe dementia, recent musculoskeletal injuries, or terminal illness. All participants provided written informed consent. The study was approved by the Ethical Committee of the University of Beira Interior (approval number: CE-UBI-PJ-2021-078) and follows the recommendations of the Declaration of Helsinki.

### Study design

In a crossover design, participants attended the gym for six weeks, completing twelve sessions in total (two per week). The first week involved familiarization with exercises and execution techniques. In the second week, a progressive loading test in the LP and CP was applied [15]. The remaining four weeks were dedicated to testing the pattern of repetition velocity over the set following the eccentric-concentric and concentric-only techniques. Each week, the training protocol for both exercises began with a session focused on the concentric-only technique, followed by another session employing the eccentric-concentric technique.

### Progressive loading test in the leg press and chest press until reaching One-Repetition maximum

Participants were assessed in two separate sessions: first, on the LP, and then after 48 h, on the CP. In the seated LP (L050, BH Fitness, Portugal), participants set their feet at shoulder width apart with knees flexed. The warm-up included two sets of seven and five repetitions with 20 and 30 kg, respectively [32]. The test started with a weight of 20 kg, which was increased by 10 kg over the sets. In the seated CP (L070, BH Fitness, Portugal), participants placed the handles at mid-chest, shoulders abducted with a neutral grip. The warm-up involved seven and five repetitions with 5 and 7.5 kg, and the test started with a weight of 5 kg, which was increased by 2.5 kg over the sets. Participants performed three repetitions whenever possible with the maximal intended velocity until reaching the 1RM load, with 3–5 min rest between sets [13].

### Exercise testing sessions

**Fig. 1:**
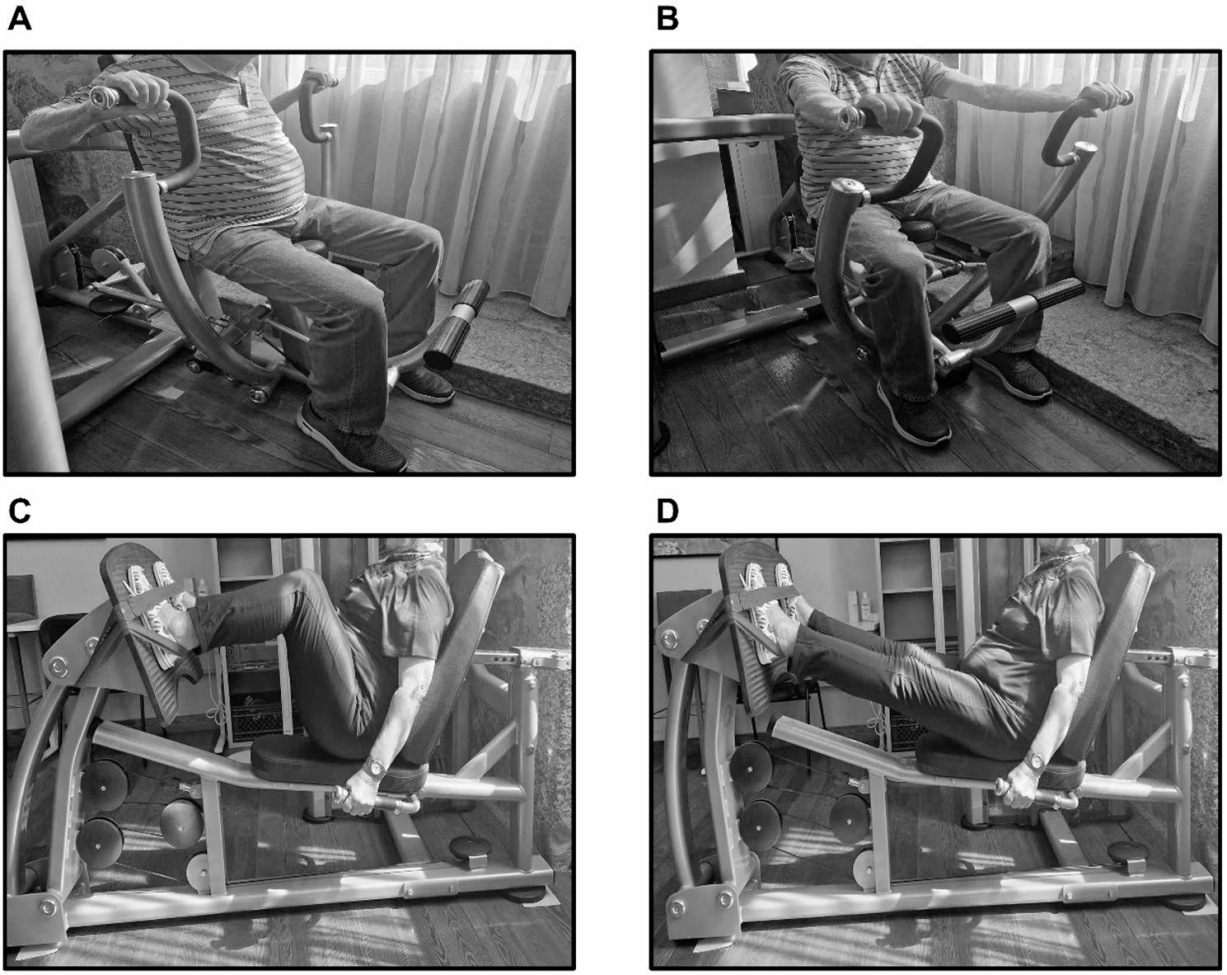
The illustration displays the starting execution techniques in the chest press (**A**: concentric-only, **B**: eccentric-concentric ) and leg press (**C**: concentric-only, **D**: eccentric-concentric)

The participants completed two sets of ten repetitions, with the relative loads assigned randomly (sequence: 40%, 60%, 70%, and 50% 1RM) across different testing sessions. The number of repetitions and relative loads were selected because they fall within the typical range recommended in resistance training guidelines for older adults [3, 33]. Each relative load was tested individually for each execution technique, resulting in eight sessions that differed only in the initial execution technique. In the first testing week, participants began with a relative load of 40%, performing the LP and CP using concentric-only technique in the first session and eccentric-concentric technique in the second. Over the next two weeks, the load changed to 60% and then 70%, using the same training techniques. In the final week, participants used a 50% load with the same methodologies. In the concentric-only technique, participants began with flexed knees (LP) or elbows (CP) at a 90-degree angle and performed a concentric action from a resting state without pre-tension. They then executed the subsequent repetitions in the usual eccentric-concentric exercise pattern (eccentric-concentric). The eccentric-concentric technique began with extended knees (LP) or elbows (CP), at an angle of 180 degrees (leg and thigh; arm and forearm), after initial assistance from the researcher lifting the LP platform and CP machine arms. After the initial adjustment, participants performed the eccentric phase followed by the concentric phase. In both conditions, the eccentric phase was performed at a controlled velocity (2 s) and the concentric phase at the maximal intended velocity. Between the eccentric and concentric phases, there was a pause of 0.5 s. In each session, participants received verbal instructions to perform eccentric repetitions slowly, followed by concentric actions executed as fast as possible [32, 34]. Two experienced coaches provided constant supervision throughout this process, ensuring proper technique and control of the timing protocol by placing their hands on the machine’s arms (CP) or the platform handles (LP). Figure 1 shows both execution techniques. A linear position transducer (Chronojump Boscosystem, version 2.3.0, Barcelona, Spain), which collects data at a sampling frequency of 1000 Hz, was used to measure MV of each repetition (i.e., the average velocity during the entire concentric phase of the movement) and control the time of the lowering phase of the movement (eccentric action) [35]. The focus was on MV to maintain consistency with previous research, as it is the most used variable in clinical contexts with older adults [13–15]. The Chronojump system is highly reliable for measuring kinematic variables in resistance machine exercises with older people [32]. The transducer’s cable was securely fixed to the right side of both resistance machines (LP and CP), ensuring consistent vertical displacement, as previously described elsewhere [32].

### Statistical analyses

The sample size estimation was performed using the statistical test “Means: Difference between two dependent means (matched pairs)” (G*Power v3.1.9.2, Dusseldorf, Germany). Results indicated a required sample size of fifteen participants for a two-tailed test with a Cohen’s d effect size of 0.80 (value based on the expected large difference between starting execution techniques in the velocity of the first repetition in both exercises, as observed in previous experiments conducted in our laboratory), an alpha level of 0.05, and 80% power. Data was first organized in Microsoft Office Excel (v2407, Microsoft 166 Corporation, Redmond, WA, USA) and then analyzed in SPSS (v27.0, IBM Corp., Armonk, NY, USA) with statistical significance set at *p* < 0.05. Normality was checked and confirmed using the Shapiro-Wilk test. Descriptive statistics are presented as mean ± standard deviation, frequencies, and percentages. The individual load-velocity profiles were generated using quadratic regressions. We modeled two regression equations for each participant: (i) the first was used to estimate the velocities (dependent variable) associated with each relative load (independent variable) and (ii) the second was used to estimate the relative loads (dependent variable) based on the fastest MV reached in the first three repetitions (independent variable) across execution techniques and exercises. With the latter individual equations, we could examine the magnitude of the relative loads associated with the velocity of the first, second, and third repetitions in both execution techniques. For each technique analyzed, the magnitude of the relative loads of the first three repetitions was calculated as the average across all participants. A 2 × 2 factorial ANOVA within-subjects, followed by post-hoc Bonferroni tests, compared the MV of all repetitions performed in the set with concentric-only vs. eccentric-concentric. The within-subject factors were repetition velocity, with 10 levels (MV of repetitions 1, 2, 3, 4, 5, 6, 7, 8, 9, and 10), and execution techniques, with two levels (concentric-only vs. eccentric-concentric). Paired-sample t-tests compared the differences between starting execution techniques (concentric-only vs. eccentric-concentric) in the fastest repetition reached in the set. Linear regressions analyzed the relationship between MV and the repetitions performed with both execution techniques in the LP and CP, allowing the analysis of the pattern of repetition velocity throughout the RT set. Finally, a within-subject repeated measures ANOVA with one factor (repetition velocity) and three levels (repetition 1 vs. repetition 2 vs. repetition 3) was used to compare the MV of the first three repetitions with the concentric-only and eccentric-concentric techniques in both exercises. The Hedge’s *g* effect size assessed the magnitude of differences (small: ≤ 0.20; moderate: 0.21–0.79; large: ≥ 0.80) [36].

## Results

### Differences between starting execution techniques in the mean velocity reached in each repetition throughout the resistance training set

**Fig. 2:**
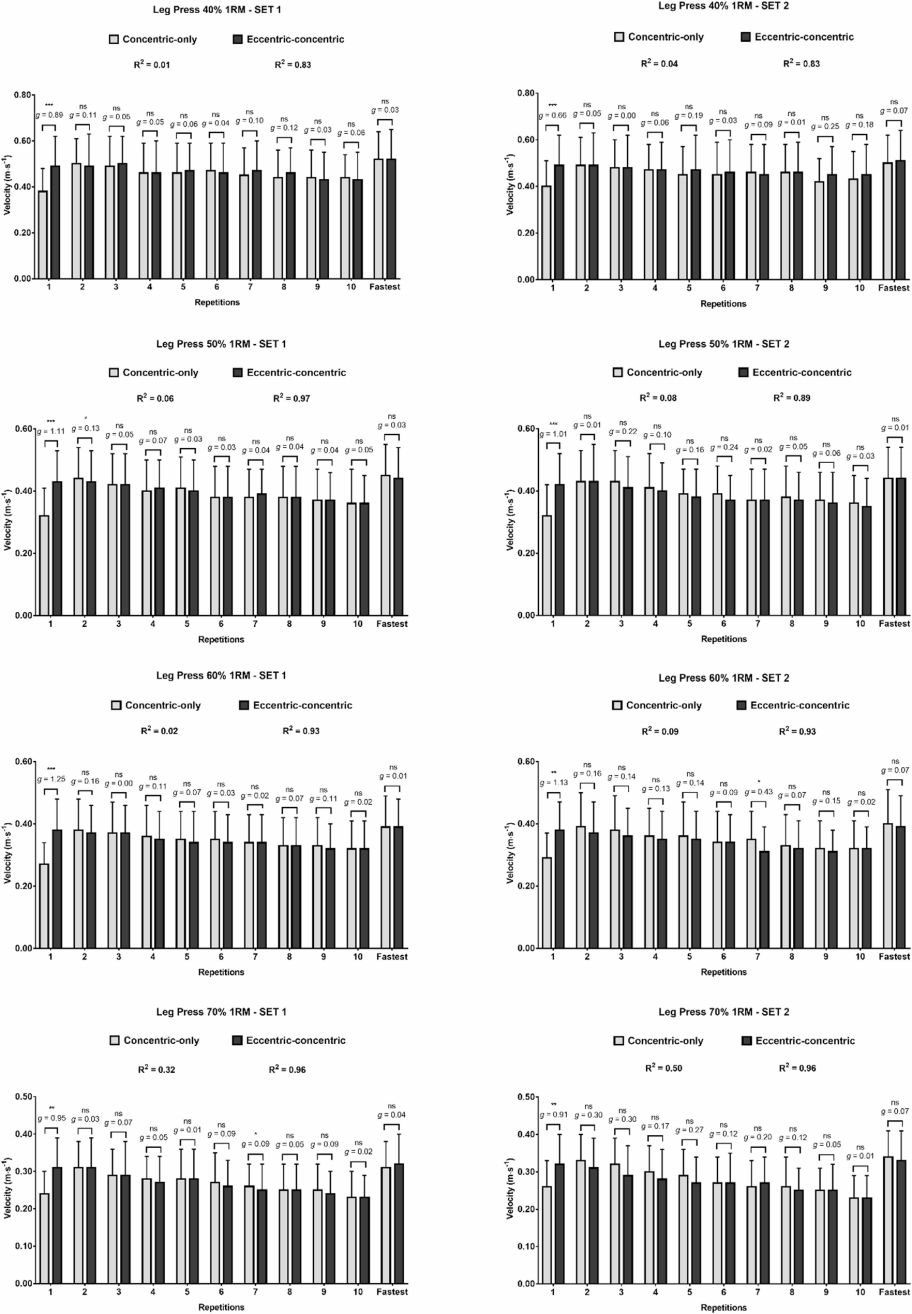
Velocity differences between execution conditions in the leg press in each repetition performed. ns, not significant; *, *p* < 0.05; **, *p* < 0.01; ***, *p* < 0.001

In the LP, moderate to large significant differences between starting execution techniques were observed for the first repetition’s velocity across all relative loads and sets, with the eccentric-concentric technique producing higher MV values than the concentric-only technique (average difference considering all observations: 0.09 ± 0.07 m·s^− 1^; *p* < 0.001; *g*: 0.84) (Fig. 2). Velocity differences between starting execution techniques were non-significant for almost all subsequent repetitions, except for the small differences in the second repetition at 50% 1RM (set 1) and seventh repetition at 70% 1RM (set 2), and the moderate difference in the seventh repetition at 60% 1RM (set 2). No differences were found between techniques when comparing the fastest repetition reached in the set (all *p* > 0.05). Regression analysis of the pattern of repetition velocity over the set in the LP revealed R² values ranging between 0.01 and 0.50 for the concentric-only technique and between 0.83 and 0.97 for the eccentric-concentric technique.

**Fig. 3:**
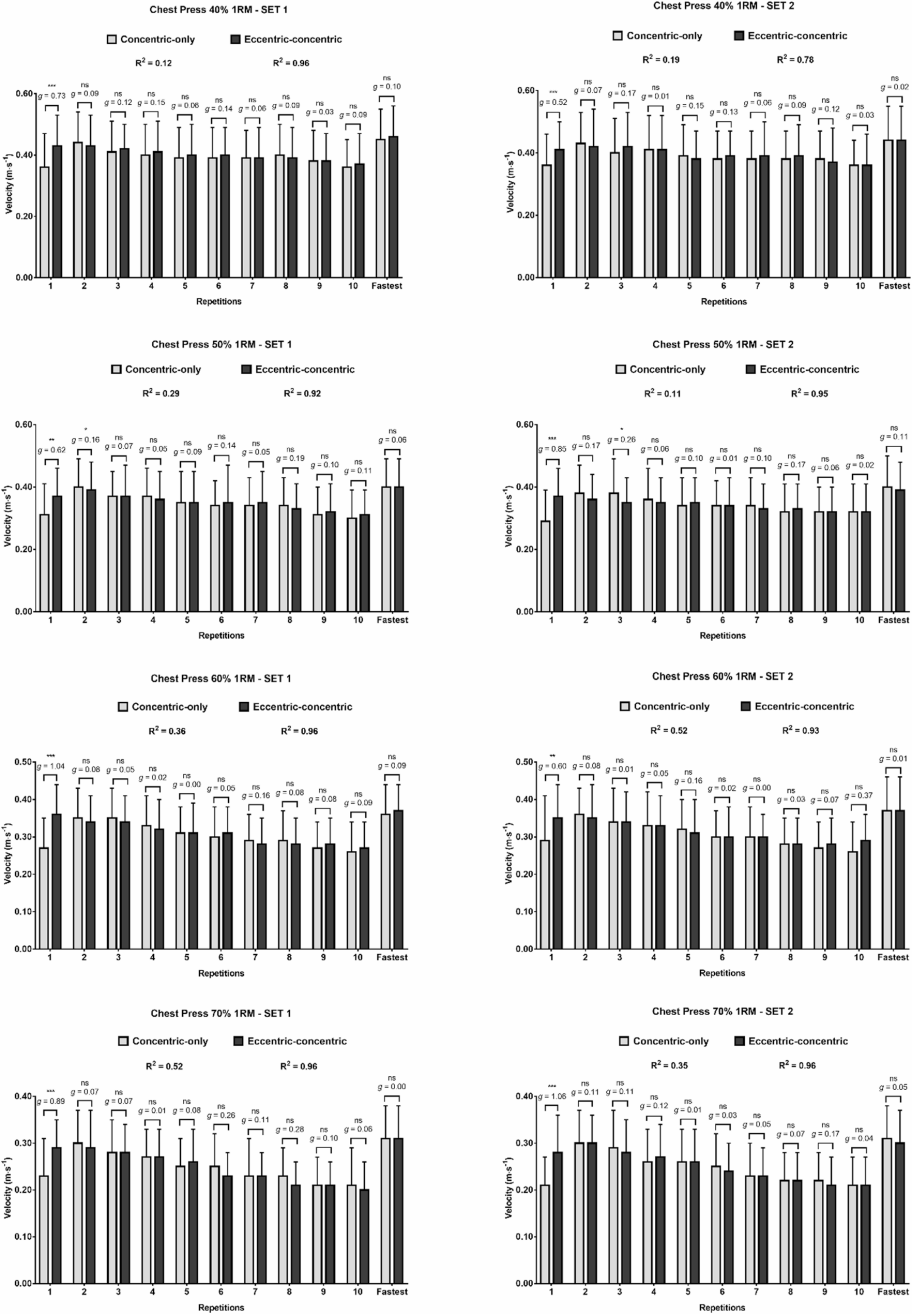
Velocity differences between execution conditions in the chest press in each repetition performed. ns, not significant; *, *p* < 0.05; **, *p* < 0.01; ***, *p* < 0.001

In the CP, moderate to large significant differences between starting execution techniques were found, with the eccentric-concentric technique achieving higher velocities in the first repetition across all loads and sets compared to the concentric-only technique (average difference considering all observations: 0.07 ± 0.06 m·s^− 1^; *p* < 0.001; *g* = 0.70) (Fig. 3). Velocity differences between starting execution techniques were non-significant for almost all subsequent repetitions, except for the small differences in the second repetition at 50% 1RM (set 1) and the moderate difference in the third repetition at 50% 1RM (set 2). No differences were found between starting execution techniques when comparing the fastest repetition reached in the set (all *p* > 0.05). Regression analysis of the pattern of repetition velocity over the set in the CP indicated R² values ranging between 0.11 and 0.52 for the concentric-only technique and between 0.78 and 0.96 for the eccentric-concentric technique.

### Analysis of the repetitions during which the fastest mean velocity was achieved most frequently using the eccentric-concentric and concentric-only starting techniques

In the LP, the fastest repetition was reached in the second repetition 65% of the time when starting with the concentric-only technique, followed by the third (28%) and first (7%) repetitions (Table 1). In contrast, the first repetition was the fastest 47% of the time for the eccentric-concentric technique, followed by the second (39%) and third (14%) repetitions. In the CP, the second repetition was the fastest 70% of the time when starting with the concentric-only technique, while with the eccentric-concentric technique, the first and second repetitions were equally the fastest at about 40% of the time (Table 1).

**Table 1:** Fastest repetition reached in the first three repetitions using both starting execution techniques

	LEG PRESS				CHEST PRESS			
	Fastest Rep	Rep 1	Rep 2	Rep 3	Fastest Rep	Rep 1	Rep 2	Rep 3
	(*n*)	(%)	(%)	(%)	(*n*)	(%)	(%)	(%)
**Concentric-only**								
40% 1RM S1	2.2 ± 0.5	5.6	**72.2**	22.2	2.2 ± 0.4	0.0	**83.3**	16.7
40% 1RM S2	2.1 ± 0.8	22.2	**44.4**	33.3	2.0 ± 0.5	11.1	**77.8**	11.1
50% 1RM S1	2.1 ± 0.3	0.0	**88.2**	11.8	2.1 ± 0.4	5.6	**83.3**	11.1
50% 1RM S2	2.5 ± 0.5	0.0	47.1	**52.9**	2.3 ± 0.6	5.6	**55.6**	38.9
60% 1RM S1	2.1 ± 0.3	0.0	**87.5**	12.5	2.5 ± 0.5	0.0	**52.9**	47.1
60% 1RM S2	2.3 ± 0.6	6.3	**56.3**	37.5	2.1 ± 0.6	11.8	**64.7**	23.5
70% 1RM S1	2.3 ± 0.5	0.0	**72.2**	27.8	2.2 ± 0.5	5.6	**66.7**	27.8
70% 1RM S2	2.1 ± 0.7	17.6	**58.8**	23.5	2.3 ± 0.5	0.0	**72.2**	27.8
All loads/sets	2.2 ± 0.5	6.6	**65.7**	27.7	2.2 ± 0.5	4.9	**69.7**	25.4
**Eccentric-concentric**								
40% 1RM S1	1.8 ± 0.8	**38.9**	**38.9**	22.2	1.8 ± 0.7	33.3	**50.0**	16.7
40% 1RM S2	2.0 ± 0.7	22.2	**55.6**	22.2	2.1 ± 0.8	27.8	**38.9**	33.3
50% 1RM S1	1.6 ± 0.8	**55.6**	27.8	16.7	2.0 ± 0.8	**33.3**	**33.3**	33.3
50% 1RM S2	1.6 ± 0.6	**50.0**	44.4	5.6	1.4 ± 0.6	**61.1**	33.3	5.6
60% 1RM S1	1.8 ± 0.8	**47.1**	29.4	23.5	1.7 ± 0.8	**52.9**	23.5	23.5
60% 1RM S2	1.6 ± 0.7	**47.1**	41.2	11.8	1.6 ± 0.7	**47.1**	41.2	11.8
70% 1RM S1	1.5 ± 0.6	**52.9**	41.2	5.9	1.7 ± 0.6	35.3	**58.8**	5.9
70% 1RM S2	1.4 ± 0.6	**64.7**	29.4	5.9	1.8 ± 0.6	29.4	**58.8**	11.8
All loads/sets	1.7 ± 0.7	**47.1**	38.6	14.3	1.8 ± 0.7	40.0	**42.1**	17.9

Bold values indicate the repetition during which the fastest mean velocity was achieved most frequently. S: set

### Comparison of the mean velocity reached in the first three repetitions using the eccentric-concentric and concentric-only starting techniques

In the LP, a significant MV difference of 0.10 ± 0.07 m·s^− 1^ (*p* < 0.001 for all loads and sets combined) between the first and second repetitions was observed with the concentric-only technique (Table 2). In contrast, starting with the eccentric-concentric technique resulted in a non-significant difference (0.00 ± 0.04 m·s^− 1^, *p* > 0.05 for all loads and sets combined) between the first and second repetition. Participants lifted an average relative load about 15% higher than the scheduled load in the first repetition (∼ 70% 1RM vs. 55% 1RM) with the concentric-only technique, while the difference with the eccentric-concentric technique was practically negligible (∼ 56% 1RM vs. 55% 1RM).

**Table 2:** Velocity differences in the first three repetitions performed with concentric-only and eccentric-concentric techniques in the leg press

	Velocity of the first three repetitions and associated relative loads						Velocity differences between repetitions					
	First repetition		Second repetition		Third repetition		Rep 1 vs. Rep 2		Rep 1 vs. Rep 3		Rep 2 vs. Rep 3	
	(m·s^-1^)	(% 1RM)	(m·s^-1^)	(% 1RM)	(m·s^-1^)	(% 1RM)	(m·s^-1^)	(*g*)	(m·s^-1^)	(*g*)	(m·s^-1^)	(*g*)
**Concentric-only**												
40% 1RM S1	0.38 ± 0.10	58.4 ± 14.2	0.50 ± 0.11	39.7 ± 7.7	0.49 ± 0.13	41.6 ± 8.0	-0.12 ± 0.08*	1.12	-0.11 ± 0.07*	0.93	0.01 ± 0.04	0.11
40% 1RM S2	0.40 ± 0.11	55.4 ± 15.2	0.49 ± 0.12	42.9 ± 12.8	0.48 ± 0.12	43.8 ± 11.4	-0.09 ± 0.09*	0.71	**-0.07 ± 0.09**ǂ	0.61	0.01 ± 0.06	0.11
50% 1RM S1	0.32 ± 0.09	70.1 ± 10.3	0.44 ± 0.10	49.8 ± 4.7	0.42 ± 0.10	53.0 ± 5.1	-0.12 ± 0.07*	1.21	-0.10 ± 0.06*	1.04	**0.02 ± 0.03#**	0.17
50% 1RM S2	0.32 ± 0.10	69.9 ± 10.6	0.43 ± 0.10	51.8 ± 7.2	0.43 ± 0.10	51.2 ± 6.8	-0.10 ± 0.06*	1.04	-0.10 ± 0.06*	1.05	0.00 ± 0.04	0.01
60% 1RM S1	0.27 ± 0.07	78.1 ± 9.0	0.38 ± 0.10	59.9 ± 4.0	0.37 ± 0.10	62.9 ± 7.1	-0.11 ± 0.07*	1.29	-0.10 ± 0.07*	1.14	0.01 ± 0.02	0.12
60% 1RM S2	0.29 ± 0.08	76.2 ± 7.8	0.39 ± 0.11	59.4 ± 6.7	0.38 ± 0.11	62.4 ± 8.9	-0.11 ± 0.07*	1.11	-0.09 ± 0.07*	0.93	0.02 ± 0.03	0.14
70% 1RM S1	0.24 ± 0.06	83.8 ± 8.9	0.31 ± 0.07	72.3 ± 3.3	0.29 ± 0.07	75.5 ± 5.7	-0.07 ± 0.05*	1.01	**-0.05 ± 0.06**ǂ	0.79	**0.02 ± 0.03#**	0.23
70% 1RM S2	0.26 ± 0.07	81.6 ± 10.9	0.33 ± 0.07	69.3 ± 4.9	0.32 ± 0.07	72.0 ± 4.0	-0.07 ± 0.07*	1.05	**-0.06 ± 0.06**ǂ	0.83	**0.01 ± 0.02#**	0.20
All loads/sets	0.31 ± 0.10	71.5 ± 14.7	0.41 ± 0.12	55.5 ± 13.1	0.40 ± 0.12	57.7 ± 13.9	-0.10 ± 0.07*	0.90	-0.09 ± 0.07*	0.77	0.01 ± 0.04*	0.11
**Eccentric-concentric**												
40% 1RM S1	0.49 ± 0.13	41.9 ± 10.9	0.49 ± 0.14	41.4 ± 12.8	0.50 ± 0.12	40.6 ± 6.6	0.00 ± 0.06	0.03	-0.01 ± 0.05	0.10	-0.01 ± 0.05	0.06
40% 1RM S2	0.49 ± 0.13	43.2 ± 9.5	0.49 ± 0.14	41.8 ± 10.9	0.48 ± 0.14	44.0 ± 9.2	-0.01 ± 0.05	0.07	0.01 ± 0.06	0.07	0.02 ± 0.05	0.13
50% 1RM S1	0.43 ± 0.10	51.9 ± 6.7	0.43 ± 0.10	52.2 ± 3.8	0.42 ± 0.10	53.5 ± 5.4	0.00 ± 0.03	0.03	0.01 ± 0.04	0.12	0.01 ± 0.02	0.09
50% 1RM S2	0.42 ± 0.10	53.0 ± 6.4	0.43 ± 0.12	53.9 ± 12.4	0.41 ± 0.10	55.3 ± 5.4	0.00 ± 0.04	0.03	0.02 ± 0.04	0.18	**0.02 ± 0.04#**	0.20
60% 1RM S1	0.38 ± 0.10	60.3 ± 4.4	0.37 ± 0.09	62.2 ± 8.0	0.37 ± 0.09	60.9 ± 5.0	0.01 ± 0.03	0.12	0.01 ± 0.02	0.09	0.00 ± 0.02	0.03
60% 1RM S2	0.38 ± 0.09	59.7 ± 7.1	0.37 ± 0.10	61.9 ± 8.3	0.36 ± 0.09	63.1 ± 6.7	0.01 ± 0.03	0.09	**0.02 ± 0.03#**	0.23	0.01 ± 0.03	0.13
70% 1RM S1	0.31 ± 0.08	72.8 ± 4.2	0.31 ± 0.08	72.6 ± 4.2	0.29 ± 0.09	76.8 ± 5.2	0.00 ± 0.02	0.02	**0.02 ± 0.03#**	0.25	**0.02 ± 0.03#**	0.24
70% 1RM S2	0.32 ± 0.08	69.1 ± 6.8	0.31 ± 0.08	72.2 ± 6.2	0.29 ± 0.08	75.4 ± 4.6	0.02 ± 0.04	0.21	**0.03 ± 0.03**ǂ	0.40	0.01 ± 0.04	0.19
All loads/sets	0.40 ± 0.12	56.2 ± 12.7	0.40 ± 0.13	57.0 ± 14.4	0.39 ± 0.12	58.4 ± 13.8	0.00 ± 0.04	0.02	0.01 ± 0.04*	0.11	**0.01 ± 0.04**ǂ	0.09

Data are presented as mean ± standard deviation. Bold values denote significant differences. S, set. g, Hedge’s g effect size. * Denotes *p* < 0.001; ǂ Denotes *p* < 0.01; # Denotes *p* < 0.05

In the CP, a significant MV difference of 0.08 ± 0.07 m·s^− 1^ (*p* < 0.001 for all loads and sets combined) between the first and second repetitions was observed in the concentric-only technique (Table 3). In contrast, beginning with the eccentric-concentric technique showed a non-significant difference (0.00 ± 0.04 m·s^− 1^, *p* > 0.05 for all loads and sets combined) between the first and second repetition. Participants lifted nearly 20% more than the scheduled load in the first repetition with the concentric-only technique (∼ 75% 1RM vs. 55% 1RM) and about 5% more with the eccentric-concentric technique (∼ 60% 1RM vs. 55% 1RM).

**Table 3:** Velocity differences in the first three repetitions performed with concentric-only and eccentric-concentric techniques in the chest press

	Velocity of the first three repetitions and associated relative loads						Velocity differences between repetitions					
	First repetition		Second repetition		Third repetition		Rep 1 vs. Rep 2		Rep 1 vs. Rep 3		Rep 2 vs. Rep 3	
	(m·s^-1^)	(% 1RM)	(m·s^-1^)	(% 1RM)	(m·s^-1^)	(% 1RM)	(m·s^-1^)	(g)	(m·s^-1^)	(g)	(m·s^-1^)	(g)
**Concentric-only**												
40% 1RM S1	0.36 ± 0.11	60.3 ± 13.3	0.44 ± 0.10	41.1 ± 9.0	0.41 ± 0.10	48.9 ± 11.0	-0.09 ± 0.07*	0.82	**-0.05 ± 0.08**ǂ	0.49	**0.04 ± 0.04**ǂ	0.35
40% 1RM S2	0.36 ± 0.10	60.3 ± 13.8	0.43 ± 0.10	44.0 ± 10.9	0.40 ± 0.11	52.3 ± 13.9	-0.07 ± 0.07*	0.68	**-0.04 ± 0.07#**	0.38	**0.03 ± 0.05#**	0.28
50% 1RM S1	0.31 ± 0.10	70.6 ± 15.3	0.40 ± 0.09	51.4 ± 8.0	0.37 ± 0.08	58.0 ± 8.5	-0.09 ± 0.07*	0.91	**-0.06 ± 0.08**ǂ	0.63	0.03 ± 0.03*	0.35
50% 1RM S2	0.29 ± 0.10	75.9 ± 13.6	0.38 ± 0.09	56.3 ± 7.5	0.38 ± 0.11	57.7 ± 10.6	-0.09 ± 0.05*	0.89	-0.09 ± 0.07*	0.83	0.00 ± 0.05	0.02
60% 1RM S1	0.27 ± 0.08	77.8 ± 12.5	0.35 ± 0.08	62.1 ± 6.8	0.35 ± 0.08	63.0 ± 7.4	-0.08 ± 0.06*	0.93	-0.07 ± 0.06*	0.91	0.00 ± 0.03	0.02
60% 1RM S2	0.29 ± 0.12	76.6 ± 18.2	0.36 ± 0.07	59.9 ± 8.1	0.34 ± 0.09	64.8 ± 11.6	**-0.07 ± 0.08**ǂ	0.67	**-0.05 ± 0.09#**	0.44	**0.02 ± 0.04#**	0.25
70% 1RM S1	0.23 ± 0.08	87.4 ± 12.3	0.30 ± 0.07	73.7 ± 6.9	0.28 ± 0.07	76.8 ± 8.6	-0.07 ± 0.06*	0.96	**-0.05 ± 0.06**ǂ	0.74	**0.02 ± 0.03#**	0.23
70% 1RM S2	0.21 ± 0.06	90.0 ± 9.4	0.30 ± 0.07	72.3 ± 7.9	0.29 ± 0.08	76.2 ± 9.9	-0.09 ± 0.07*	1.39	-0.08 ± 0.07*	1.08	**0.02 ± 0.03#**	0.21
All loads/sets	0.29 ± 0.11	74.8 ± 16.9	0.37 ± 0.10	57.6 ± 13.9	0.35 ± 0.10	62.2 ± 14.0	-0.08 ± 0.07*	0.79	-0.06 ± 0.07*	0.60	0.02 ± 0.04*	0.19
**Eccentric-concentric**												
40% 1RM S1	0.43 ± 0.10	42.6 ± 15.7	0.43 ± 0.10	41.6 ± 13.0	0.42 ± 0.08	43.6 ± 10.7	0.00 ± 0.05	0.01	0.02 ± 0.06	0.16	0.02 ± 0.04	0.16
40% 1RM S2	0.41 ± 0.09	46.8 ± 9.7	0.42 ± 0.12	46.3 ± 10.5	0.42 ± 0.11	47.6 ± 15.7	-0.01 ± 0.05	0.13	-0.01 ± 0.04	0.10	0.00 ± 0.04	0.03
50% 1RM S1	0.37 ± 0.09	57.9 ± 8.5	0.39 ± 0.09	54.0 ± 7.2	0.37 ± 0.10	57.5 ± 12.1	-0.01 ± 0.04	0.15	0.00 ± 0.05	0.03	0.01 ± 0.04	0.12
50% 1RM S2	0.37 ± 0.09	57.0 ± 9.1	0.36 ± 0.08	59.6 ± 8.4	0.35 ± 0.08	61.6 ± 9.1	0.01 ± 0.05	0.14	0.02 ± 0.04#	0.24	0.01 ± 0.03	0.12
60% 1RM S1	0.36 ± 0.08	61.7 ± 7.7	0.34 ± 0.07	64.7 ± 6.5	0.34 ± 0.07	65.2 ± 8.7	0.02 ± 0.04	0.21	0.02 ± 0.03	0.20	0.00 ± 0.04	0.00
60% 1RM S2	0.35 ± 0.09	63.4 ± 8.9	0.35 ± 0.09	64.6 ± 11.5	0.34 ± 0.08	67.1 ± 9.7	0.00 ± 0.03	0.03	0.02 ± 0.03	0.19	0.01 ± 0.03	0.15
70% 1RM S1	0.29 ± 0.06	75.1 ± 7.8	0.29 ± 0.08	74.6 ± 9.8	0.28 ± 0.06	77.8 ± 9.5	0.00 ± 0.04	0.03	0.01 ± 0.02#	0.21	0.02 ± 0.04	0.22
70% 1RM S2	0.28 ± 0.08	76.4 ± 10.7	0.30 ± 0.06	73.5 ± 8.5	0.28 ± 0.07	76.1 ± 7.7	-0.01 ± 0.04	0.16	0.01 ± 0.04	0.08	0.02 ± 0.02*	0.26
All loads/sets	0.36 ± 0.10	59.9 ± 14.9	0.36 ± 0.10	59.6 ± 14.7	0.35 ± 0.10	61.8 ± 15.5	0.00 ± 0.04	0.01	0.01 ± 0.04#	0.09	0.01 ± 0.04*	0.11

Data are presented as mean ± standard deviation. Bold values denote significant differences. S, set. g, Hedge’s g effect size. * Denotes *p* < 0.001; ǂ Denotes *p* < 0.01; # Denotes *p* < 0.05

## Discussion

This study investigated the impact of the initial execution techniques – eccentric-concentric vs. concentric-only – on the pattern of repetition velocity throughout the RT set in the LP and CP among older adults. The main findings of this study indicated: (i) moderate to large significant differences between starting execution techniques for the first repetition’s velocity across all relative loads, with the eccentric-concentric technique allowing higher velocity outputs than the concentric-only technique; (ii) no differences existed between conditions when comparing the fastest repetition reached in the set in both exercises; (iii) the eccentric-concentric technique enabled a more stable pattern of repetition velocity over the set in both exercises, as evidenced by the higher R² values; (iv) starting the LP and CP with the concentric-only technique contributed to reaching the fastest MV in the second repetition more frequently, while the eccentric-concentric technique contributed to achieving the fastest MV in the first repetition almost half of the time. These results corroborate our study hypothesis by demonstrating that adopting an initial concentric-only technique in the LP and CP decreases the velocity in the first repetition. Furthermore, there were no significant differences between execution techniques concerning the fastest MV completed within the set.

Velocity-monitored RT in older adults enables a precise prescription of the relative load and individualizes the training volume during each session [13–15]. Following this methodological procedure, coaches can adjust training loads in real-time, manage daily fatigue, and offer well-informed guidance to practitioners [10, 19, 37]. The fastest repetition (usually the first one) in each set is a crucial variable because it allows coaches to determine the relative load (associated with a specific velocity value) and control the level of effort, mainly through the control of a predetermined intra-set velocity loss [11]. Research with strength-trained men showed that the first repetition was the fastest 75% of the time in short sets, while in long sets, it was only 37% of the time [12]. Depending on the methodologies used, the selected variable (MV or peak velocity) and the number of repetitions performed (short or long sets), the first repetition may not necessarily be the fastest [12]. For example, in geriatric settings, Marques et al. [14, 15] showed that, on average, the second repetition was the fastest in the set when using the initial concentric-only technique in the LP and CP. Similarly, the current research corroborates these findings by showing that the initial concentric-only technique contributed to achieving the highest MV outputs in the second repetition compared to the first, with the latter reporting very low values. The velocity differences in the first repetition compared to the second repetition in LP and CP ranged from 0.08 to 0.10 m·s^− 1^ (for all loads and sets combined). These results confirm that lower velocity outputs are expected in the first repetition when using the starting concentric-only technique in resistance machines (LP and CP), as it is more challenging to overcome the “sticking point” at the start of the movement [21, 38, 39].

Through the individual load-velocity profiles, we estimated the associated relative loads in the first, second, and third repetitions in both execution techniques. When examining the first repetition in the concentric-only technique, it can be observed that the magnitude of the relative load increases by almost 15–20% beyond the programmed load. This estimated increase in the relative load following the concentric-only technique was due to the lower velocity output reached in the first repetition. This result can be attributed to the greater challenge of overcoming the “sticking point” region at the beginning of the movement when performing the exercise on resistance machines [21, 22]. These results also suggest that older adults using this technique can perceive a significantly higher level of effort at the start of the exercise; however, it is important to note that they attain the prescribed relative load by the second or third repetition. This increase in the magnitude of the relative load in the first repetition might have negative consequences in older adults, as it can prevent them from executing the repetition when training with high relative loads (e.g., with relative loads of 75% 1RM, the first repetition can represent ∼ 90% 1RM) and may still predispose the older individual to a greater risk of muscle injury. Additionally, using the first repetition in the set as a reference when applying the concentric-only technique can unintentionally increase training volume, especially if intra-set velocity loss is taken into account. For example, if we consider a velocity of 0.40 m·s^− 1^ in the first repetition (reference) for a velocity loss of 20%, the participant will end the set when the velocity drops to 0.32 m·s^− 1^. However, if the participant reaches a velocity of 0.50 m·s^− 1^ in the second repetition, but the velocity loss is already set at 0.32 m·s^− 1^, it means that the participant will need to perform more repetitions to reach the target velocity of 0.32 m·s^− 1^. Therefore, given the implicit effort required when starting with the concentric-only technique in resistance machines, such as the LP and CP, adopting a different starting execution technique may be preferable in the older population (e.g., the eccentric-concentric technique). This approach requires some assistance and access to a machine that facilitates the participant placing their legs and arms in the required position.

Importantly, our results showed that when using the eccentric-concentric technique, there were no differences in velocity between the first and second repetitions, thus respecting the programmed relative load. Additionally, the eccentric-concentric technique enabled a more stable pattern of repetition velocity throughout the set, as indicated by the high levels of the coefficient of determination. The observed results can be fundamentally attributed to the preceding eccentric phase, which effectively stimulates pre-tension in the muscles and facilitates a reduction in muscle slack, ultimately enhancing overall performance, as indicated in prior research [29]. Similar findings were reported in a study that examined lower-body performance through LP in the older population [8]. McDermott et al. [8] found that older adults exhibited higher velocity outputs (+ 15 to 54%) after a prior eccentric phase across four different relative loads (35%, 50%, 65%, and 80% 1RM). Indeed, implementing a pre-stretching phase increases the velocity of the subsequent movement (stretch-shortening cycle) [21, 24]. In our study, the eccentric phase was performed at a controlled velocity, with a 0.5-second pause in the transition between eccentric and concentric phases; such aspects may adversely affect the stretch-shortening cycle, particularly in processes that enable the storage of elastic energy and its subsequent release, as well as the enhancement of residual force [28, 31, 40]. On the other hand, the management of this pause was conducted subjectively, which may have unintentionally resulted in a rebound effect, potentially leading to an improvement in concentric action [27, 40, 41].

Although our results reinforce a velocity difference in the first repetition between execution techniques, it is essential to note that there were no differences between execution techniques for almost all subsequent repetitions performed in the set, and when comparing the fastest repetition in the set. Therefore, these findings help reinforce that, regardless of the starting execution technique used, coaches and researchers should consider the fastest MV of the set as the reference point to determine the relative intensity and velocity loss threshold, as it will be similar in both techniques.

In terms of practical applications, these results underscore the significance of adopting an initial eccentric-concentric technique in resistance machines for older adults, particularly in the LP and CP, as it enhances the likelihood of identifying the fastest repetition during the first attempt (with no differences for the second). This approach accurately identifies the relative load and facilitates a more stable pattern of repetition velocity throughout the RT set, as evidenced by the high R² values (0.78 and 0.97), yielding benefits for managing the relative velocity loss in subsequent repetitions.

This study has some limitations that need to be addressed. First, although the relative loads were randomly assigned, all participants followed the same sequence through the four different load conditions. Furthermore, no counterbalancing order was implemented among the techniques employed, with the concentric-only technique being consistently performed as the first session of the exercise testing weeks and the eccentric-concentric technique as the second. The aspects above may have contributed to an order effect, which could result in potential bias. Second, a between-day (short-term) and long-term reliability analysis would provide valuable insights regarding the consistency of results among participants over time [42]. Third, future research should ensure equal representation of women and men matched for strength to examine whether there are differences between sexes when matched for strength in their repetition velocity patterns using both execution techniques. Finally, future studies must consider selecting a broad spectrum of relative loads (20–90%) and ensure strict control over the transition time between the concentric and eccentric phases, which may provide better insights into the pattern of repetition velocity over the set with both initial execution techniques. Additionally, incorporating other exercises, particularly those that involve pulling movements (e.g., seated rows), may yield different results.

## Conclusions

This study shows that the design of velocity-monitored RT in older adults should consider two fundamental aspects when using the LP and CP exercises: the execution technique at the beginning of the movement and the reference repetition used to determine the relative intensity and relative intra-set velocity loss. In older adults, when starting with the eccentric-concentric technique, the fastest velocity is expected to occur during the first two repetitions, showing minimal differences between them. Furthermore, the initial eccentric-concentric technique also helps reduce the excessive mechanical effort associated with adopting the concentric-only technique, which may provide practical training benefits and a more stable pattern of repetition velocity throughout the RT sets in older individuals.

## Data Availability

Data will be made available by the corresponding author on a reasonable request. E-mail address: tiago.sousa@ubi.pt.

## Abbreviations

1RM: One-repetition maximum
CP: Chest press
LP: Leg press
MV: Mean velocity
RT: Resistance training
